# Supplementary figures and images for: Identification of miR136, miR155, and miR183 in Vascular Calcification in Human Peripheral Arteries
Source: Int J Mol Sci. 2025 Sep 25;26(19):9349. doi: 10.3390/ijms26199349 (PMC12525181; doi:10.3390/ijms26199349)

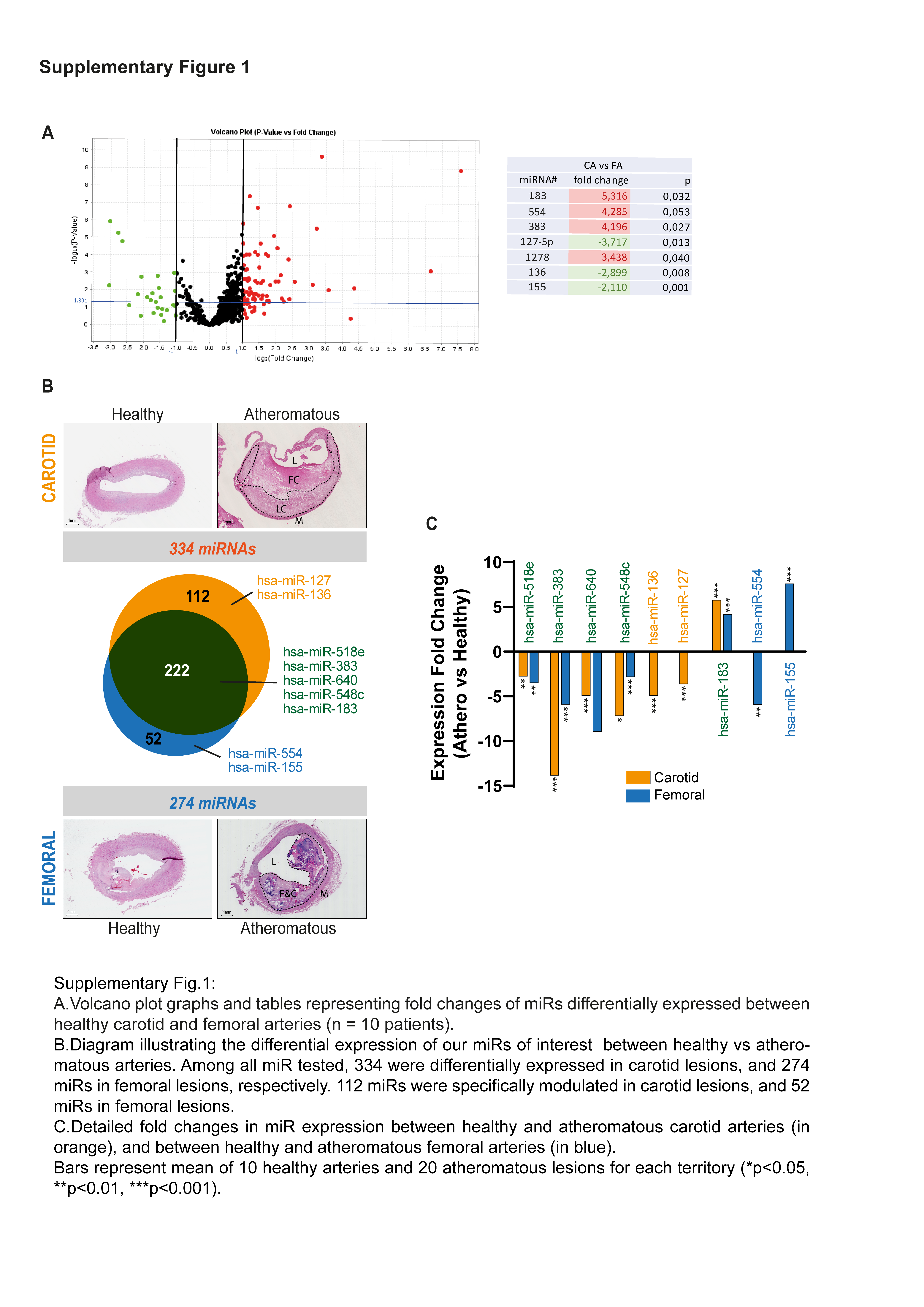

Supplement: Supplementary file 1 [file ijms-26-09349-s001.zip › Supplementary Figures/Supplementary Figure S1 projet miRs_IJMS.tif]

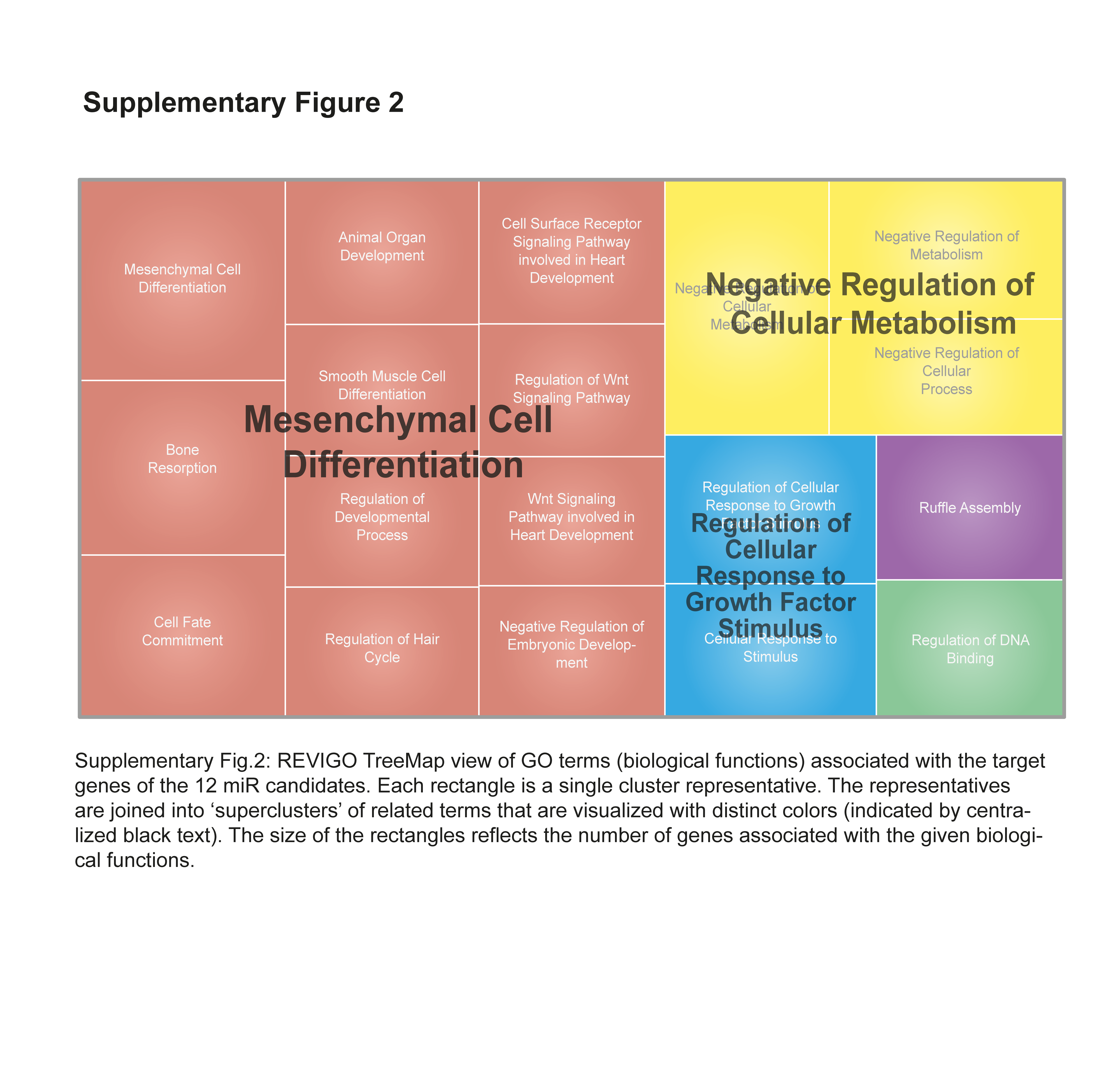

Supplement: Supplementary file 1 [file ijms-26-09349-s001.zip › Supplementary Figures/Supplementary Figure S2 projet miRs_IJMS.tif]

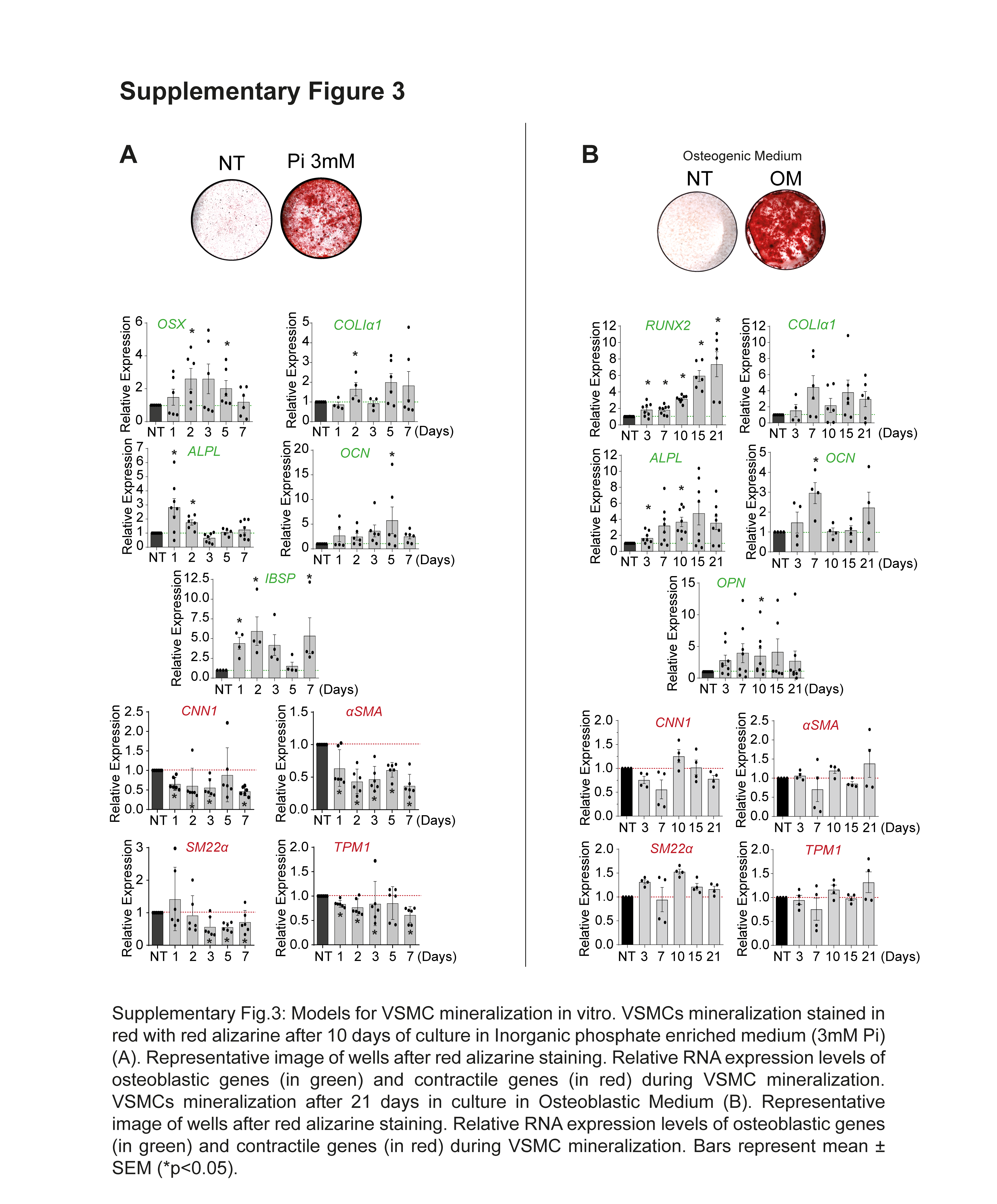

Supplement: Supplementary file 1 [file ijms-26-09349-s001.zip › Supplementary Figures/Supplementary Figure S3 projet miRs_IJMS.tif]

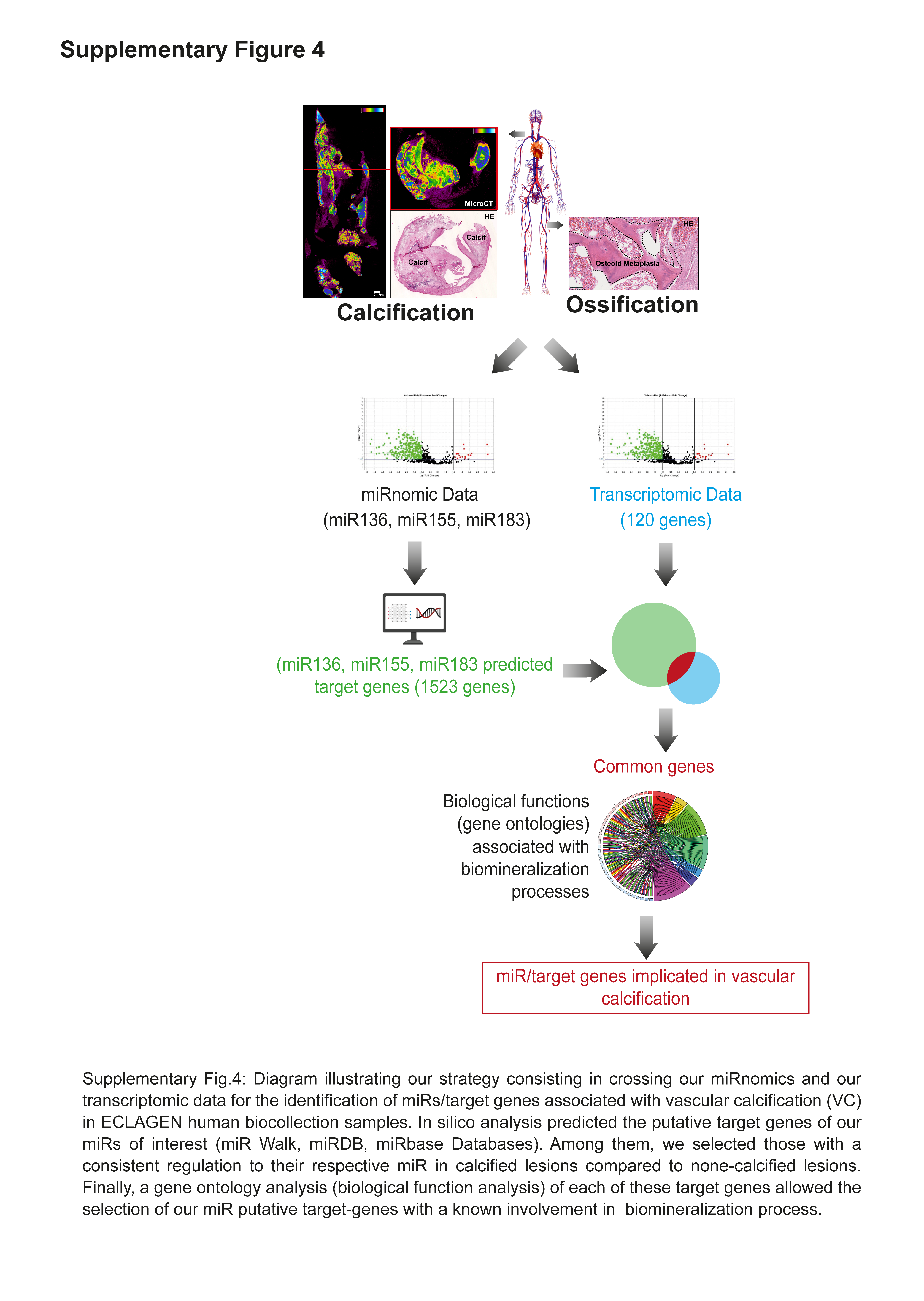

Supplement: Supplementary file 1 [file ijms-26-09349-s001.zip › Supplementary Figures/Supplementary Figure S4 projet miRs_IJMS.tif]
